# Supplementary material for: Long QT syndrome type 3 gain-of-function of Nav1.5 increases ventricular fibroblasts proliferation and pro-fibrotic factors
Source: Commun Biol. 2025 Feb 11;8:216. doi: 10.1038/s42003-025-07636-5 (PMC11814334; doi:10.1038/s42003-025-07636-5)
Supplement: Supplementary file 1 — Supplementary Information [file 42003_2025_7636_MOESM1_ESM.pdf]

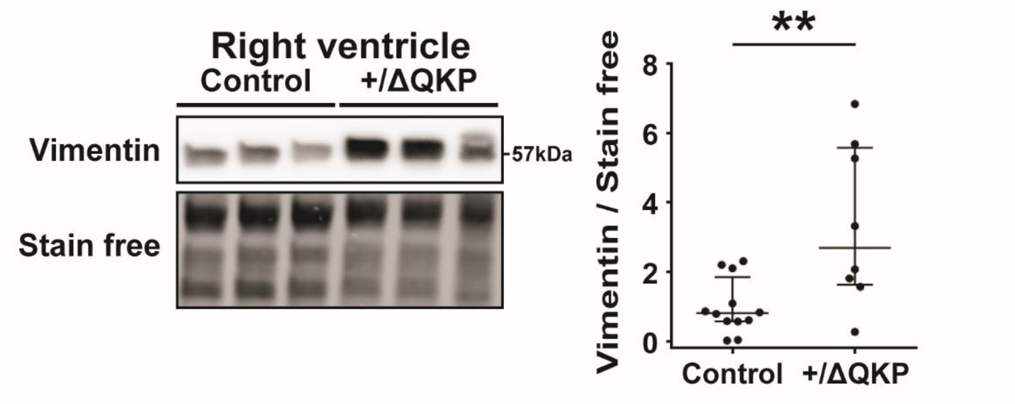

Supplementary Figure 1. Vimentin expression is increased in *Scn5a*$^{+/\Delta QKP}$ mouse right ventricle.

Representative western blot and protein expression of vimentin in right ventricle from 4-week-old control and *Scn5a*$^{+/\Delta QKP}$ mice (n = 12 & 8, respectively). Median and interquartile range are shown. Dots indicates values of each independent mouse. **, p< 0.01 (Student t-test).

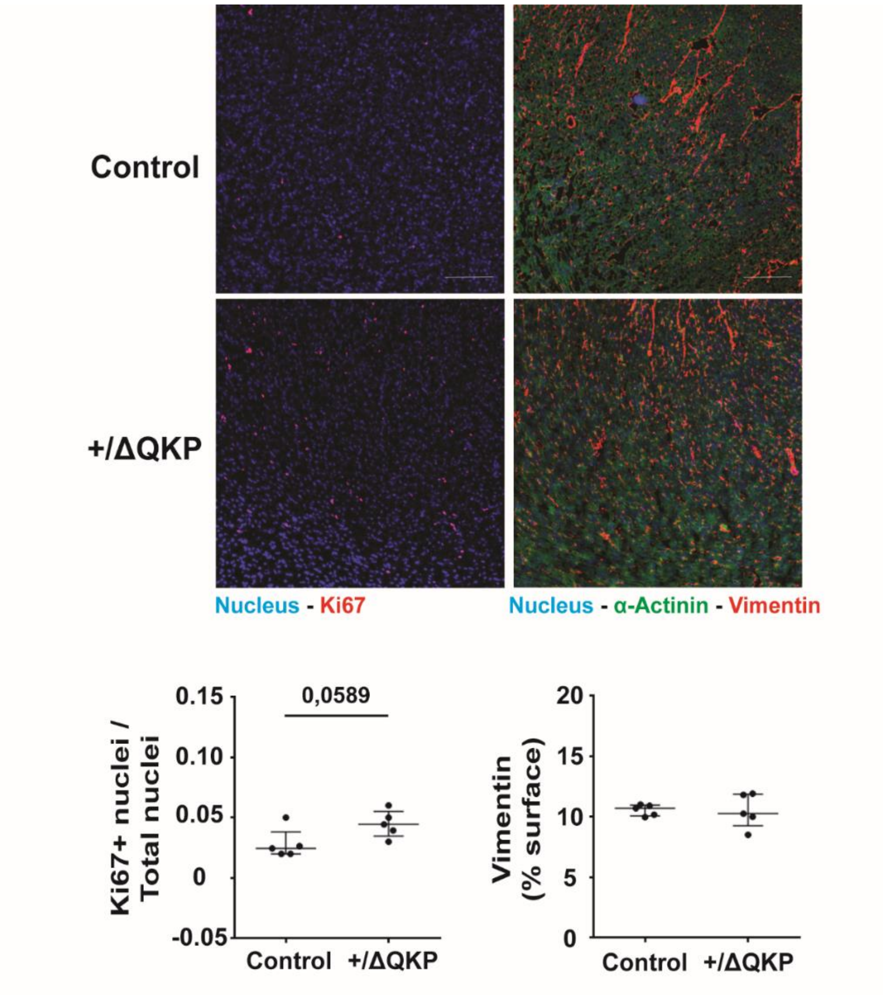

Supplementary Figure 2. Vimentin and Ki67 expression in left ventricular sections of hearts from 2-week-old control and *Scn5a*$^{+/\Delta QKP}$ mice.

Representative transverse immunostainings of vimentin (red), α-actinin (green), and Ki67 (red) in left ventricular sections of hearts from 2-week-old control and *Scn5a*$^{+/\Delta QKP}$ mice (scale bar: 50 µm and quantification of Ki67-positive nuclei and vimentin-expressing cells. Median and interquartile range are shown. Dots indicates values of each independent mouse.

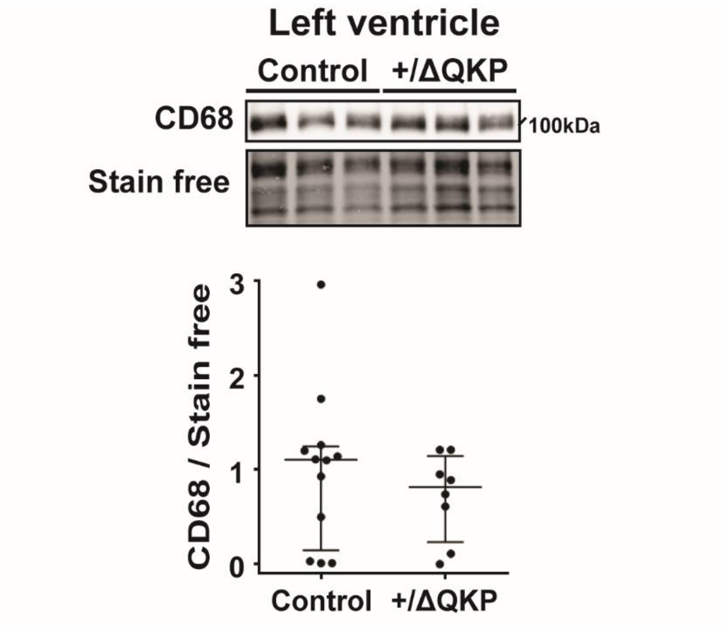

Supplementary Figure 3. Expression of CD68, a marker of macrophages, in *Scn5a*$^{+/\Delta QKP}$ mouse ventricle is unchanged.

Representative western blot and CD68 expression in 4-week-old control and *Scn5a*$^{+/\Delta QKP}$ mouse ventricular tissue (n = 12 & 8, respectively). Median and interquartile range are shown. Dots indicates values of each independent mouse.

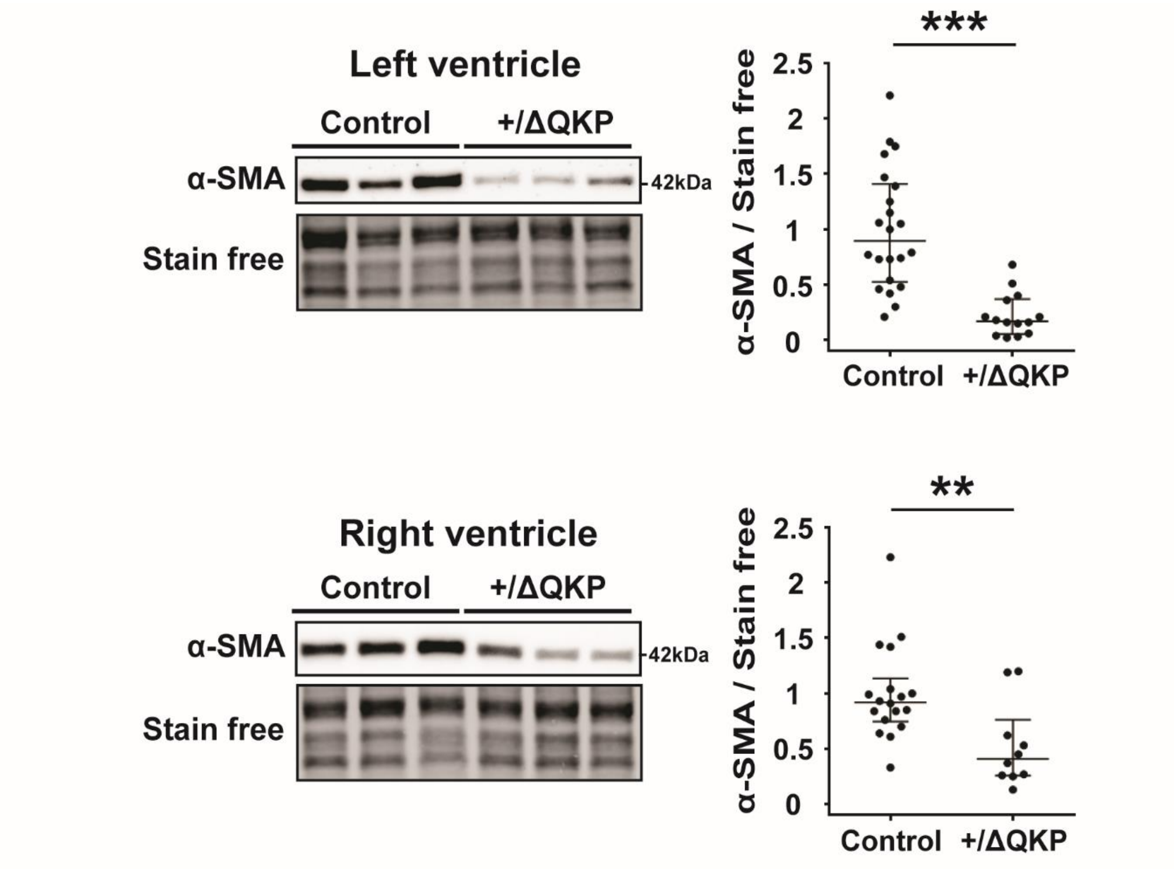

Supplementary Figure 4. α-Smooth muscle actin (α-SMA) expression is decreased in *Scn5a*$^{+/\Delta QKP}$ mouse ventricles.

Representative western blot and α-SMA expression in 4-week-old control and *Scn5a*$^{+/\Delta QKP}$ mice in left (n = 22 &14, respectively) and right (n = 18 &10, respectively) ventricles. Median and interquartile range are shown. Dots indicates values of each independent mouse. **, ***, p< 0.01 and 0.001, respectively (Mann-Whitney test).

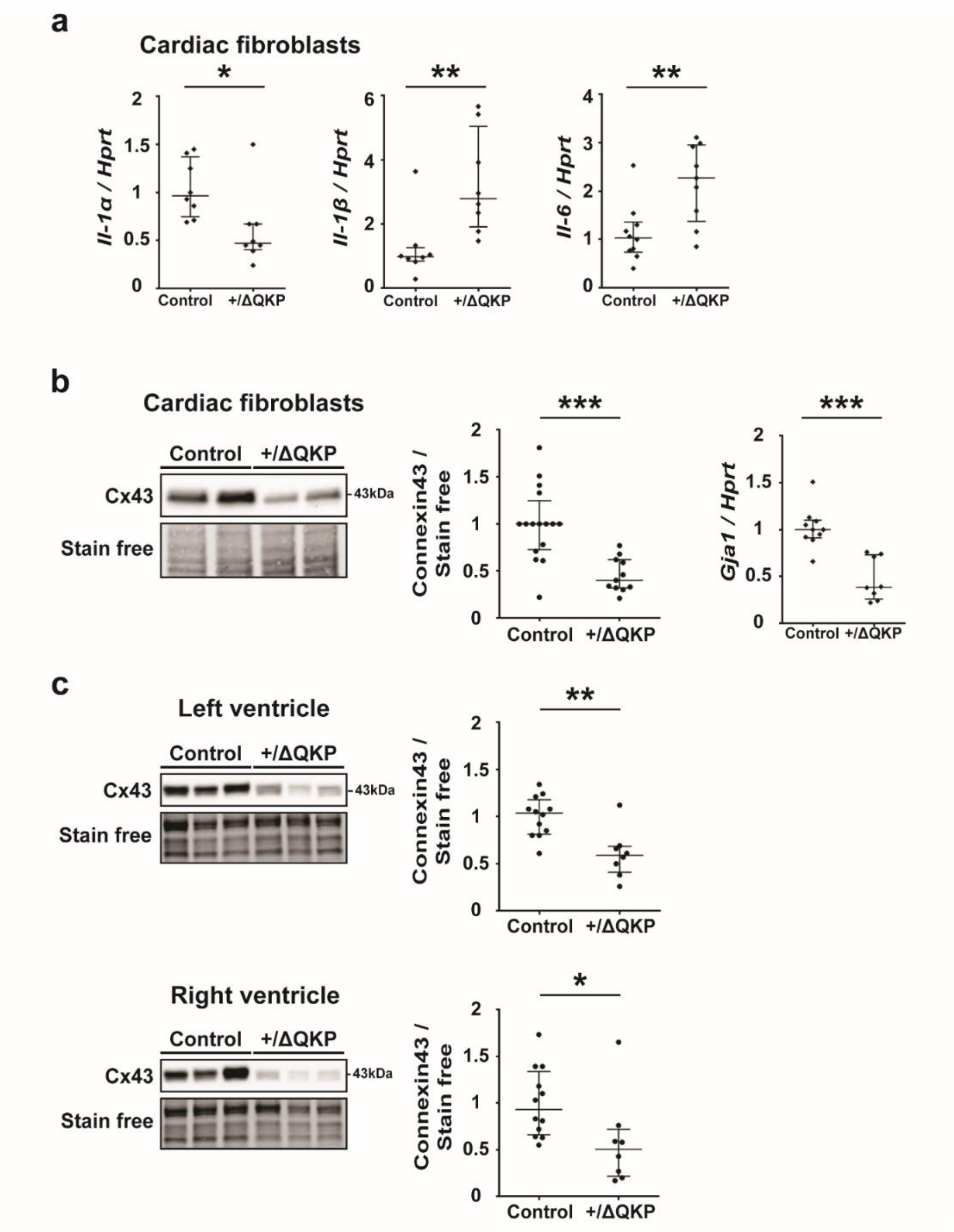

Supplementary Figure 5. Expression of interleukins *Il-1α, Il-1β, Il-6* and connexin 43 in control and *Scn5a*$^{+/\Delta QKP}$ mouse ventricular fibroblasts and expression of connexin 43 in control and *Scn5a*$^{+/\Delta QKP}$ mouse ventricles.

a *Il-1α* (n = 8, each), *Il-1β* (n = 8, each) and *Il-6* (n = 10, each) mRNA expression in fibroblasts from control and *Scn5a*$^{+/\Delta QKP}$ ventricles relative to *Hprt* and normalized to control mean value ($2^{-\Delta\Delta Ct}$). **b** Representative western blot and connexin 43 expression (Cx43; n = 16 & 11, respectively) in cardiac fibroblasts, and *Gja1* (gene encoding Cx43) mRNA expression relative to *Hprt* and normalized to control mean value ($2^{-\Delta\Delta Ct}$) (n = 10 & 8, respectively). **c** Representative western blot and Cx43 expression in left (n = 12 & 8, respectively) and right (n = 12 & 8, respectively) ventricles. From mice at 4-week-old. Median and interquartile range are shown. Dots indicates values of each independent mouse. *, **, ***, p< 0.05, 0.01 and 0.001, respectively (Mann-Whitney test).

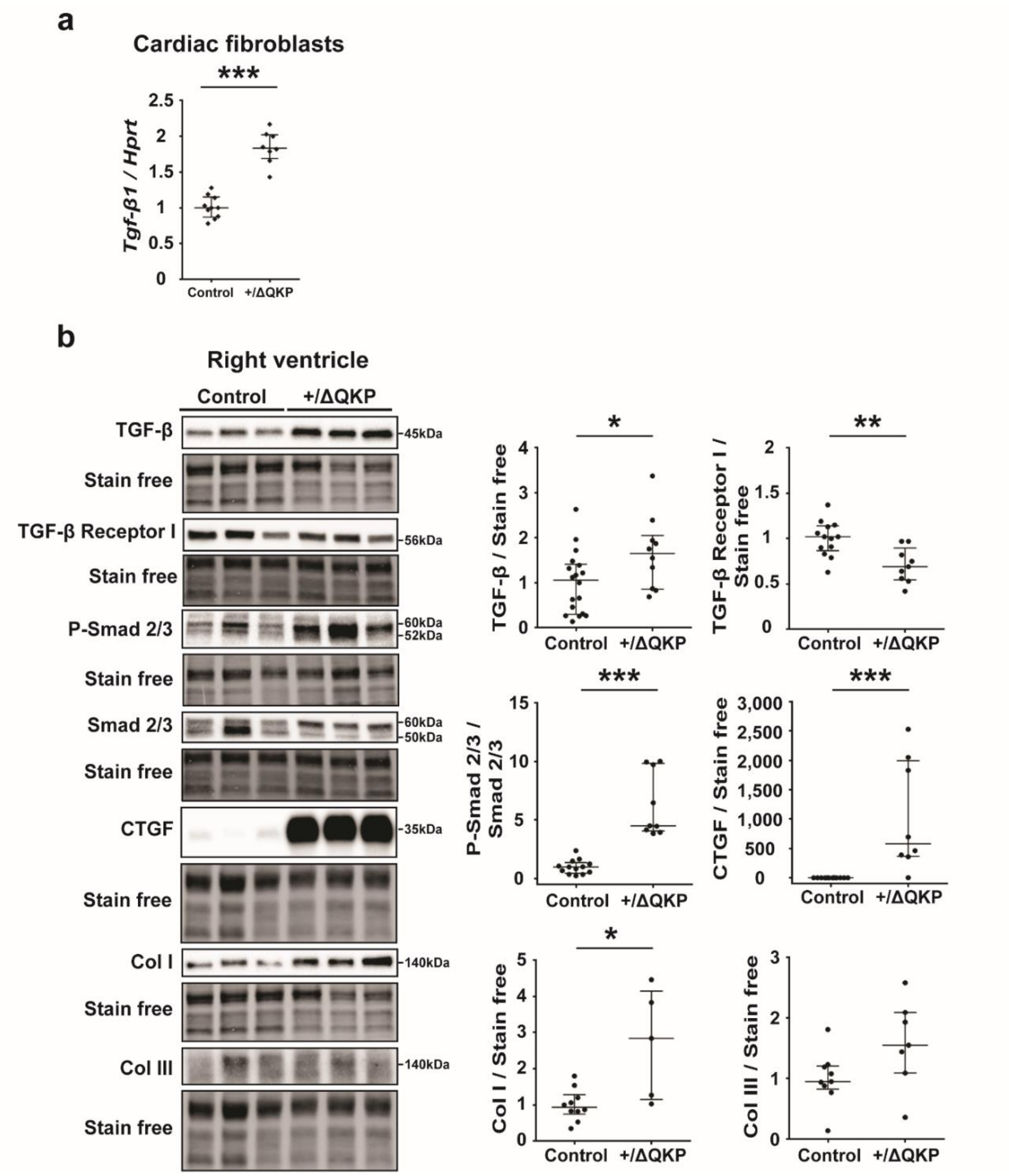

Supplementary Figure 6. Expression of transforming growth factor β (TGF-β) is increased in *Scn5a*$^{+/\Delta QKP}$ mouse right ventricle.

a *Tgf-β1* mRNA expression in fibroblasts from control and *Scn5a*$^{+/\Delta QKP}$ ventricles, relative to *Hprt*, and normalized to control mean value ($2^{-\Delta\Delta Ct}$) (n = 10 & 8, respectively). **b** Representative western blot and protein expression, in right ventricle from control and *Scn5a*$^{+/\Delta QKP}$ mice, of TGF-β (n= 18 & 10, respectively), TGF-β Receptor I (n= 13 & 9, respectively), CTGF ; n = 11 & 12, respectively), phosphorylated-Smad 2/3, Smad 2/3, and ratio (n = 13 & 8, respectively), Collagen I (Col I; n = 10 & 5, respectively) and III (Col III; n = 9 & 7, respectively). From mice at 4-week-old. Median and interquartile range are shown. Dots indicates values of each independent mouse. *, **, ***, p< 0.05, 0.01 and 0.001, respectively (Mann-Whitney test).

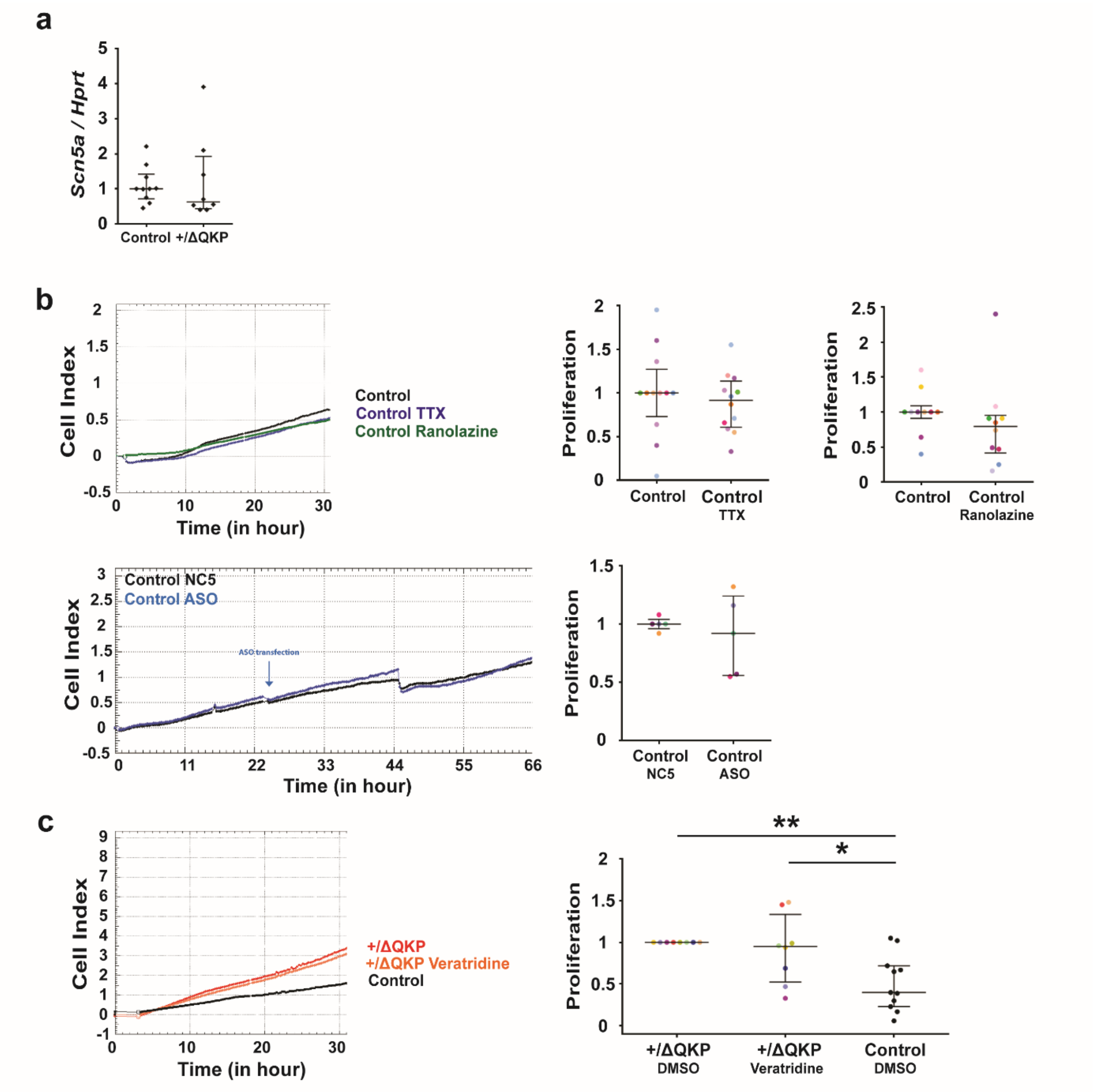

Supplementary Figure 7. TTX, ranolazine and ASO treatments do not have any effect on proliferation of control mouse ventricular fibroblasts despite Nav1.5 expression.

a *Scn5a* mRNA expression in fibroblasts from control and *Scn5a*$^{+/\Delta QKP}$ hearts, relative to *Hprt* and normalized to control mean value (n = 10 & 8, respectively). **b** Representative xCELLigence cell index time course and proliferation rate for control fibroblasts treated with 50 µM TTX (n = 12), 50 µM ranolazine (n = 10), for control fibroblasts transfected with the negative control NC5 or anti-*Scn5a* ASO (n= 5, each), and for rate for control fibroblasts (n=10), or *Scn5a*$^{+/\Delta QKP}$ fibroblasts treated with 1 µM of veratridine, or vehicle (DMSO) (n= 8, each). Data are normalized to untreated, NC5 control values, or DMSO control. Experiments performed with the same mouse are indicated by the same colour. From mice at 4-week-old. Median and interquartile range are shown. Dots indicates values of each independent mouse. *, **, $p < 0.05$ and 0.01, respectively (Kruskal-Wallis test).

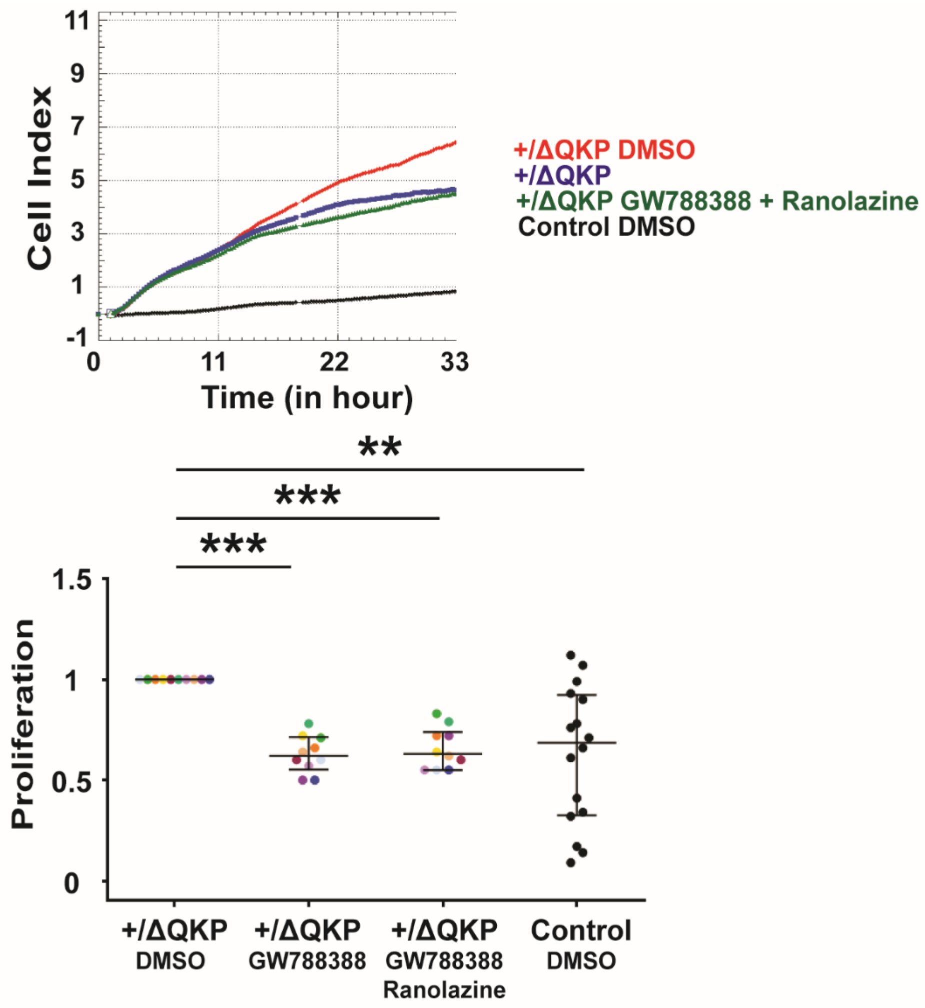

Supplementary Figure 8. GW788388 and ranolazine treatments do not have cumulative effect on proliferation a of control and *Scn5a*$^{+/\Delta QKP}$ mouse ventricular fibroblasts.

Representative xCELLigence cell index time course and proliferation rate for *Scn5a*$^{+/\Delta QKP}$ fibroblasts (n = 10) treated with vehicle (DMSO), 20 µM of GW788388 or 50 µM ranolazine + 20 µM GW788388, compared to control fibroblasts treated with vehicle (DMSO; n = 13)**.** Data are normalized to DMSO *Scn5a*$^{+/\Delta QKP}$ values. Experiments performed with fibroblasts from the same mouse are indicated by the same colour. From mice at 4-week-old. Median and interquartile range are shown. Dots indicates values of each independent mouse. **, ***: p< 0.01 and 0.001, respectively (Kruskal-Wallis test).

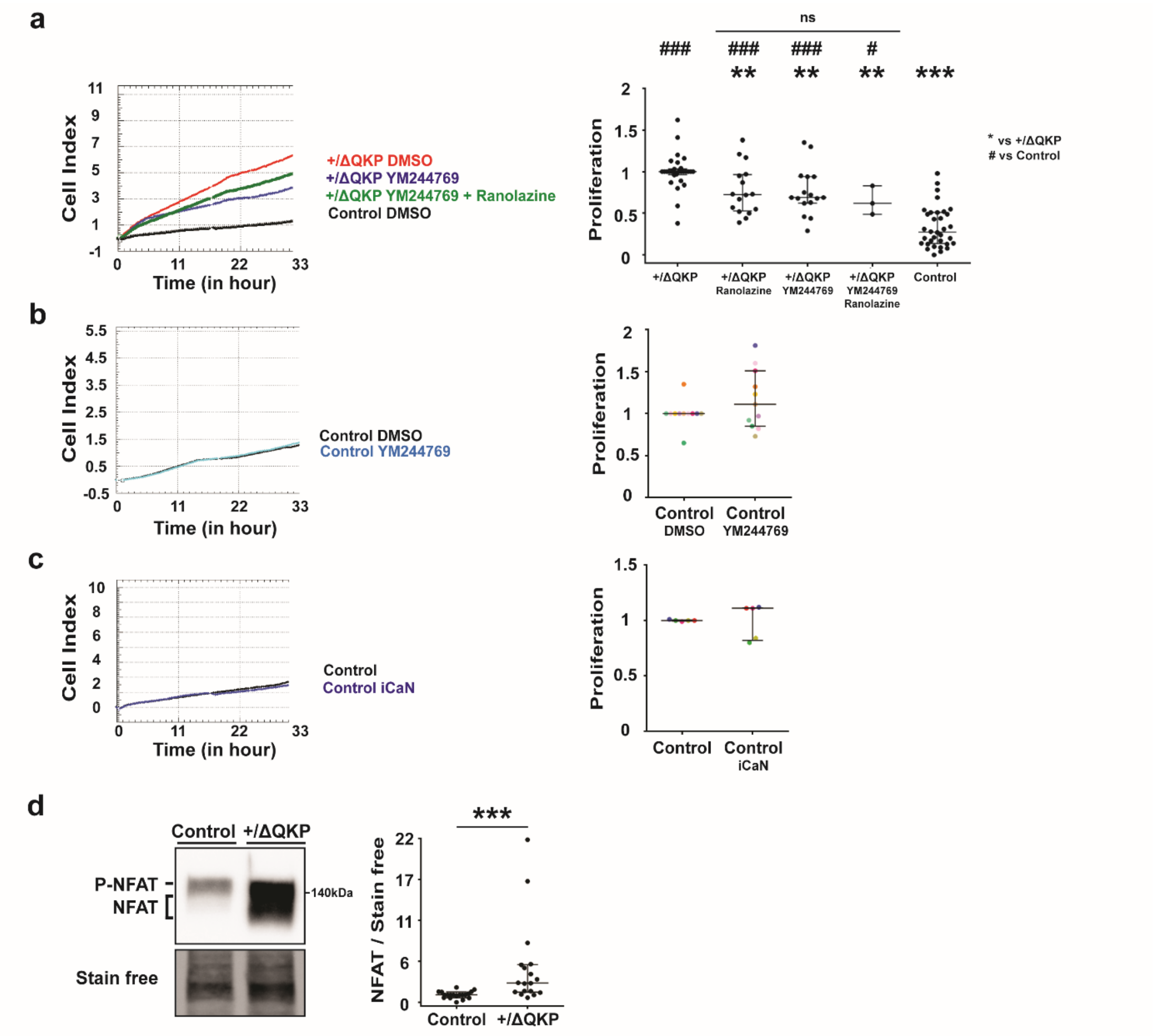

Supplementary Figure 9. YM244769 and ranolazine effects on proliferation of control and *Scn5a*$^{+/\Delta QKP}$ mouse ventricular fibroblasts.

Representative xCELLigence cell index time course and proliferation rate for **(a)** *Scn5a*$^{+/\Delta QKP}$ fibroblasts treated with 50 µM ranolazine, 10 µM YM244769, or both (n = 16, 16 & 3, respectively) or for control DMSO-treated fibroblasts (n= 34). Data are normalized to *Scn5a*$^{+/\Delta QKP}$ mean value (n = 29). Experiments performed with the same mouse are indicated by the same colour. Data as in (a) for control fibroblasts treated with 10 µM YM244769 **(b)** or 10 µM iCaN (**c**) (n = 11 & 5, respectively). Data are normalized to DMSO- or untreated control mean value. **(d)** Representative western blots and protein expression of NFAT and P-NFAT in fibroblasts from control and *Scn5a*$^{+/\Delta QKP}$ hearts (n= 19 & 18, respectively). From mice at 4-week-old. Median and interquartile range are shown. Dots indicates values of each independent mouse. *, **, ***: p< 0.05, 0.01 and 0.001, respectively (ANOVA one-way test and Mann-Whitney test).

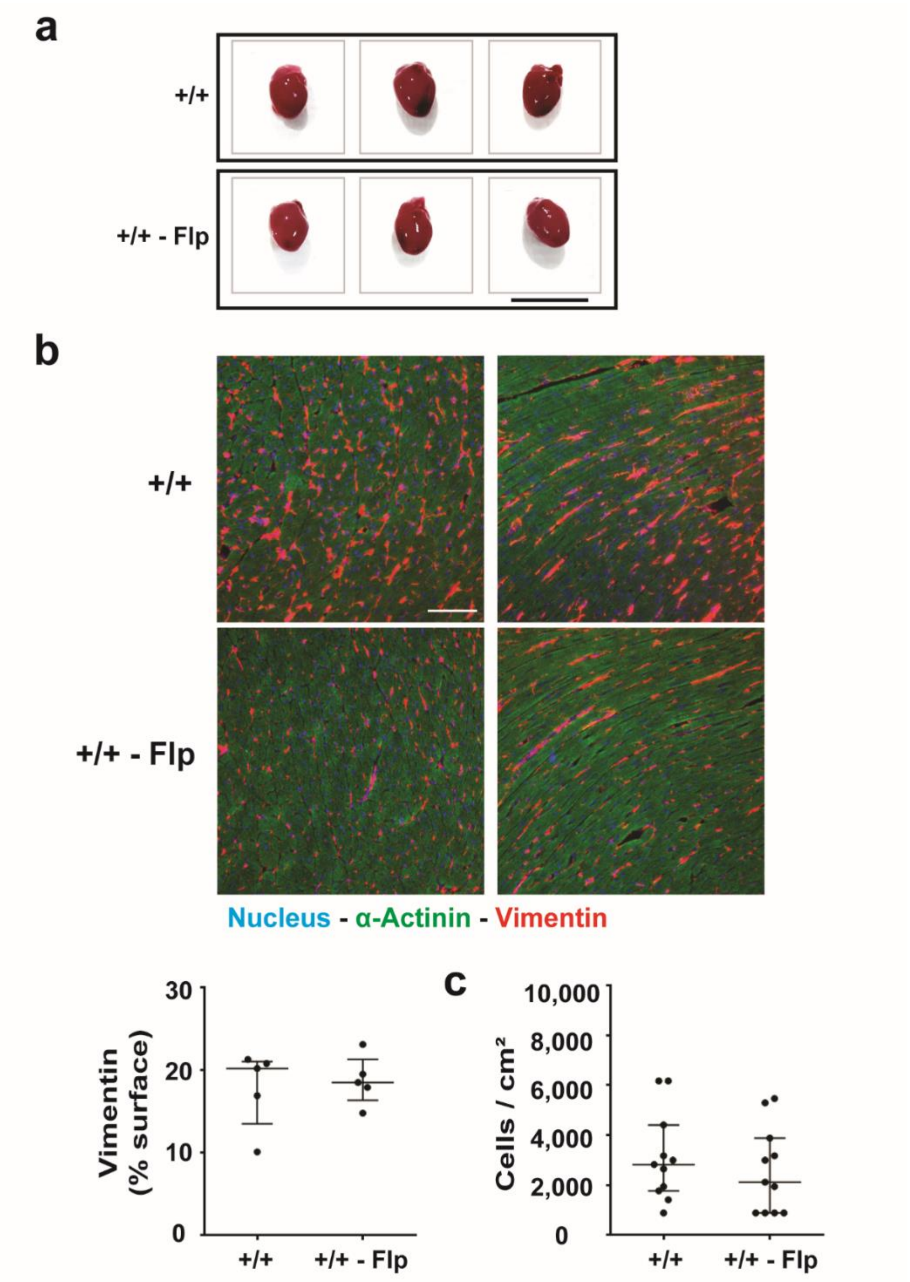

Supplementary Figure 10. *In-vivo* or *in-vitro* ventricular fibroblast abundance does not differ in S*cn5a*$^{+/+}$ and *Scn5a*$^{+/+}$*-Flp* mice.

a Representative hearts from *Scn5a*$^{+/+}$ and *Scn5a*$^{+/+}$-*Flp* mice (scale bar: 1 cm). **b** Representative transverse and longitudinal immunostainings of vimentin (red) in left ventricular sections of *Scn5a*$^{+/+}$ and *Scn5a*$^{+/+}$-*Flp* mice and percentage of vimentin-positive nuclei (n = 5, each) (scale bar: 50 µm). Cardiomyocyte marker α-actinin is stained in green. **c** Cell density of fibroblasts from *Scn5a*$^{+/+}$ and *Scn5a*$^{+/+}$-*Flp* hearts at day 5 of culture (cells/cm^2; n = 11, each). From mice at 4-week-old. Median and interquartile range are shown. Dots indicates values of each independent mouse.

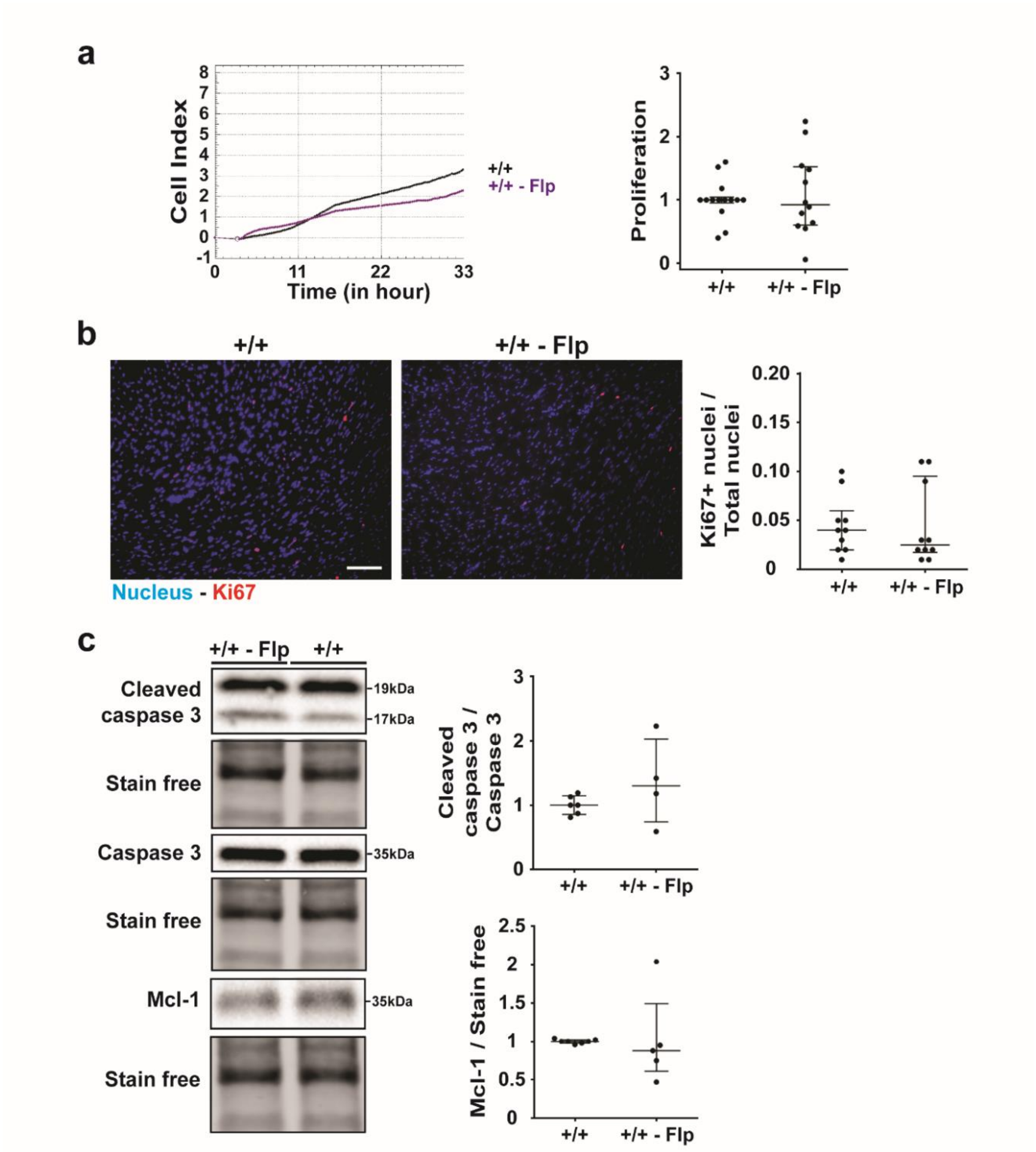

Supplementary Figure 11. Proliferation or cell survival of ventricular fibroblasts does not differ in *Scn5a*$^{+/+}$ and *Scn5a*$^{+/+}$ *-Flp* mice.

a Representative xCELLigence cell index time course and proliferation rate for fibroblasts from *Scn5a*$^{+/+}$ and *Scn5a*$^{+/+}$*-Flp* mouse ventricles (n = 14 & 12, respectively). Data are normalized to *Scn5a*$^{+/+}$ mean value. **b** Representative immunostainings of Ki67 (red) in left ventricular sections of *Scn5a*$^{+/+}$ and *Scn5a*$^{+/+}$*-Flp* mice (scale bar: 50 µm), and percentage of Ki67-positive nuclei (n = 10, each). **c** Representative western blot and protein expression in fibroblasts from *Scn5a*$^{+/+}$ and *Scn5a*$^{+/+}$*-Flp* hearts, of cleaved caspase 3 expression / caspase 3 (n = 6 & 4, respectively), and Mcl-1 (n = 7 & 5, respectively). From mice at 4-week-old. Median and interquartile range are shown. Dots indicates values of each independent mouse.

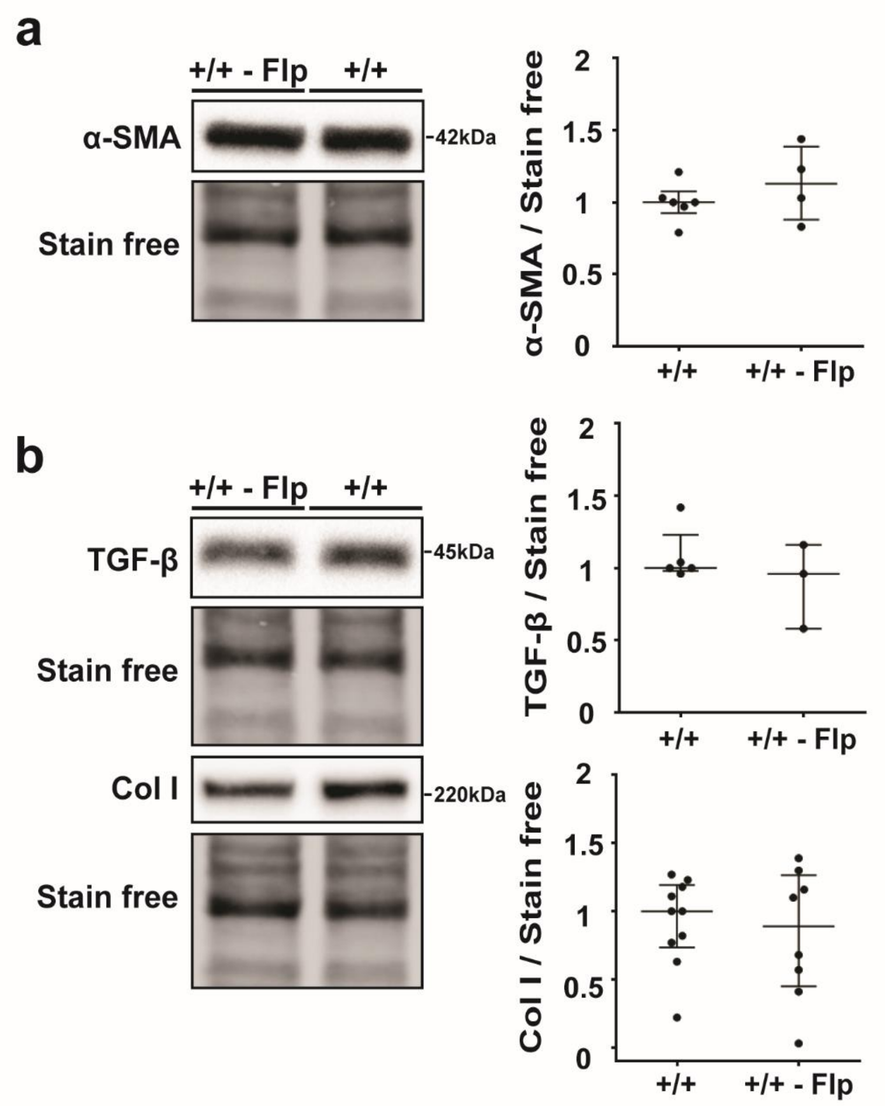

Supplementary Figure 12. Phenotype or production of ventricular fibroblasts does not differ in *Scn5a*$^{+/+}$ and *Scn5a*$^{+/+}$*-Flp* mice.

Representative western blot and protein expression in fibroblasts from *Scn5a*$^{+/+}$ and *Scn5a*$^{+/+}$*-Flp* hearts, of α-SMA (a; n = 6 and 4, respectively), TGF-β (b; n = 5 & 3, respectively) and Col I (n = 10 & 8, respectively). From mice at 4-week-old. Median and interquartile range are shown.

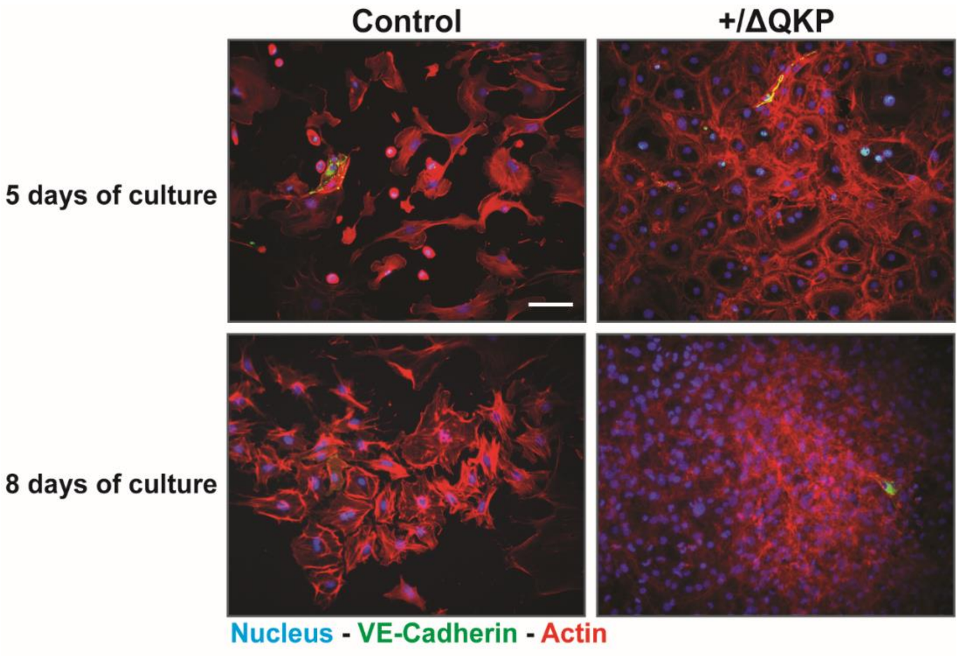

Supplementary Figure 13. Quality control of primary cultures of ventricular fibroblasts from control and *Scn5a*$^{+/\Delta QKP}$ mice.

Representative immunostainings of VE-cadherin (green) and staining of F-actin with rhodamine-phalloidin (red) in cardiac fibroblasts from control and *Scn5a*$^{+/\Delta QKP}$ mice (scale bar: 50 µm) at days 5 and 8 of culture. From mice at 4-week-old.

Supplementary Information

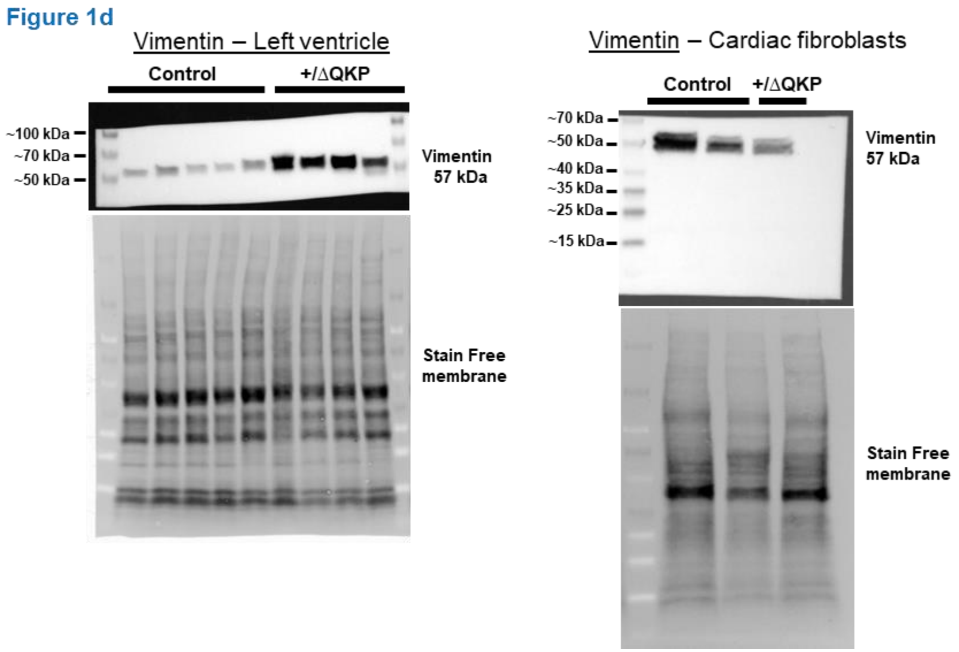

Supplementary Information 1. Uncropped and unmodified blot images from Figure 1d.

Uncropped and unmodified blot images for the representative western blots of vimentin expression in 4-week-old control and *Scn5a*$^{+/\Delta QKP}$ mouse left ventricles, and fibroblasts at day 8 of culture.

Figure 2c

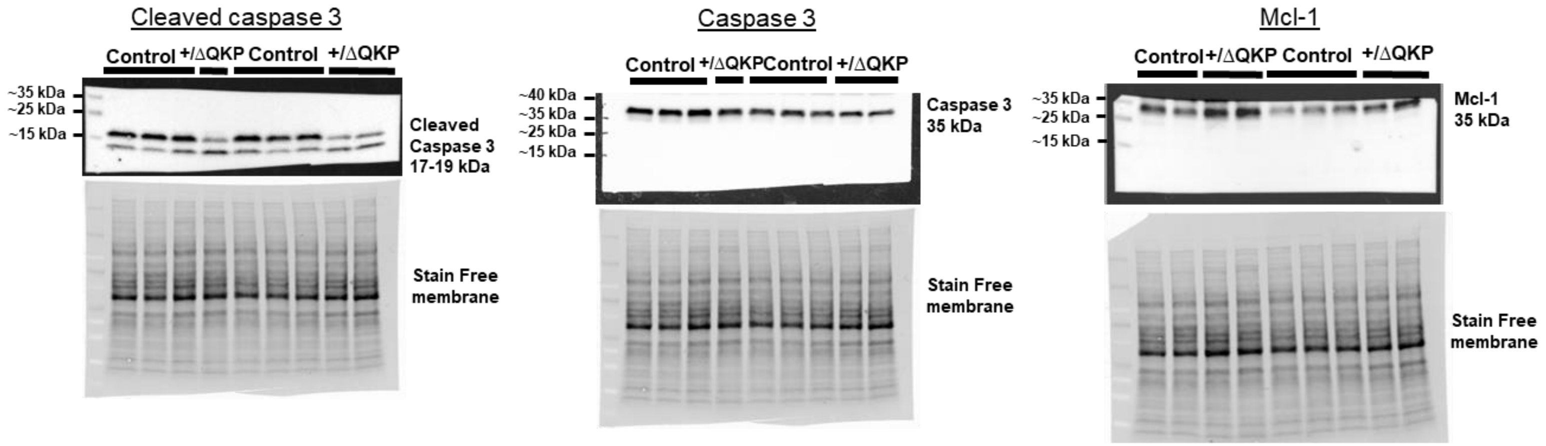

Figure 2f

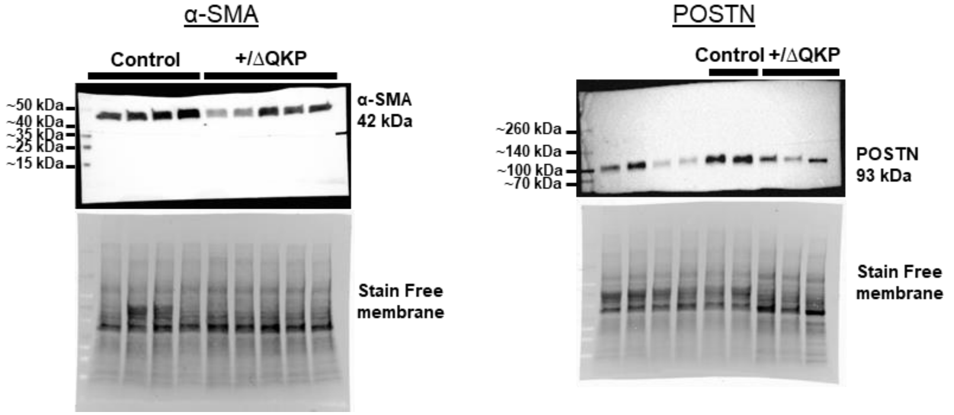

Supplementary Information 2. Uncropped and unmodified blot images from Figure 2c and 2f.

Uncropped and unmodified blot images for the representative western blots of cleaved caspase 3 / caspase 3 and Mcl-1 (Figure 2c), and of α-SMA and periostin (POSTN) (Figure 2f) in fibroblasts from control and *Scn5a*$^{+/\Delta QKP}$ hearts.

Figure 3a - Cardiac fibroblasts

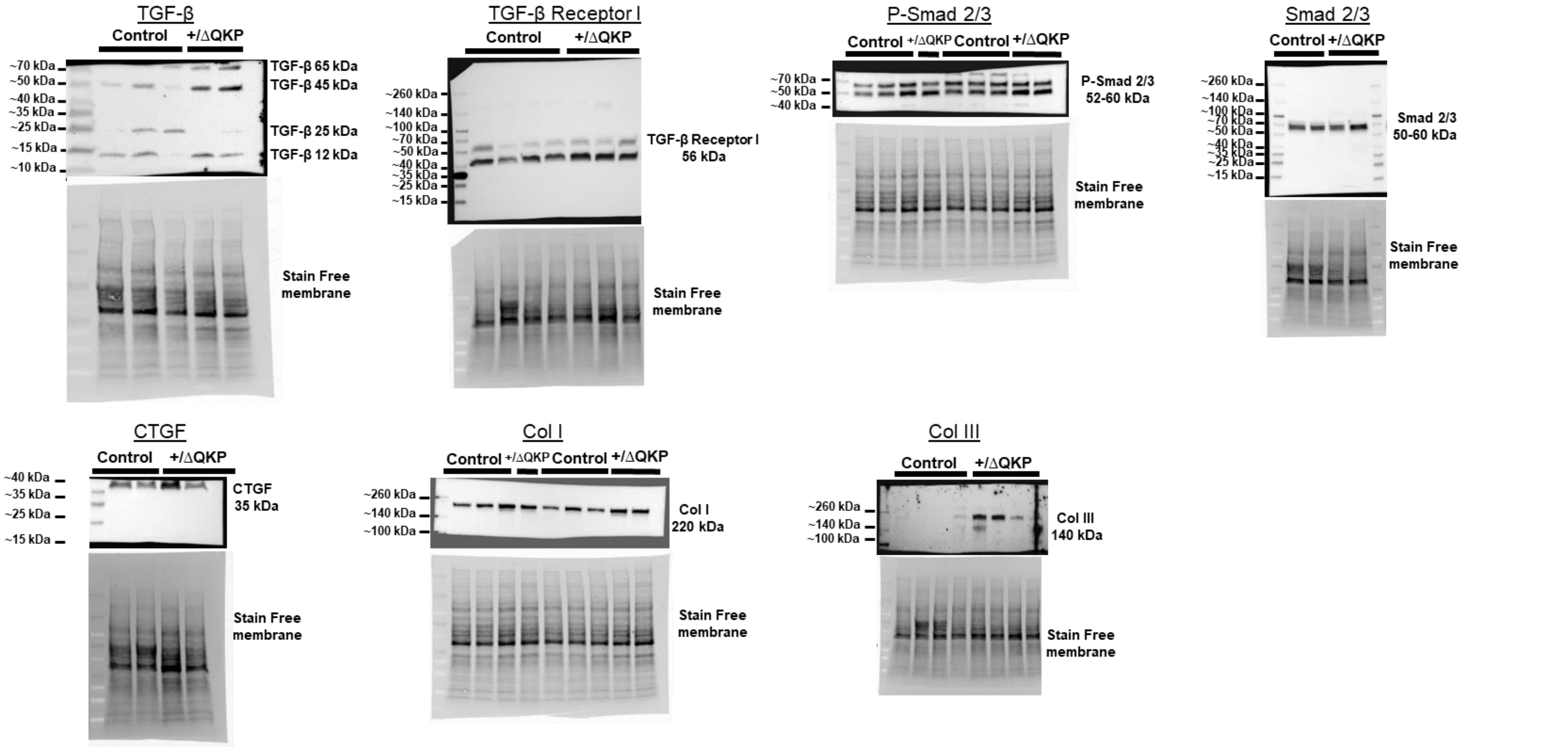

Supplementary Information 3. Uncropped and unmodified blot images from Figure 3a

Uncropped and unmodified blot images for the representative western blots of TGF-β, TGF- β receptor 1, Smad 2/3 and its phosphorylated form (P-Smad 2/3), connective tissue growth factor (CTGF), collagen I (Col I), and collagen III (Col III) in fibroblasts from control and *Scn5a*$^{+/\Delta QKP}$ hearts.

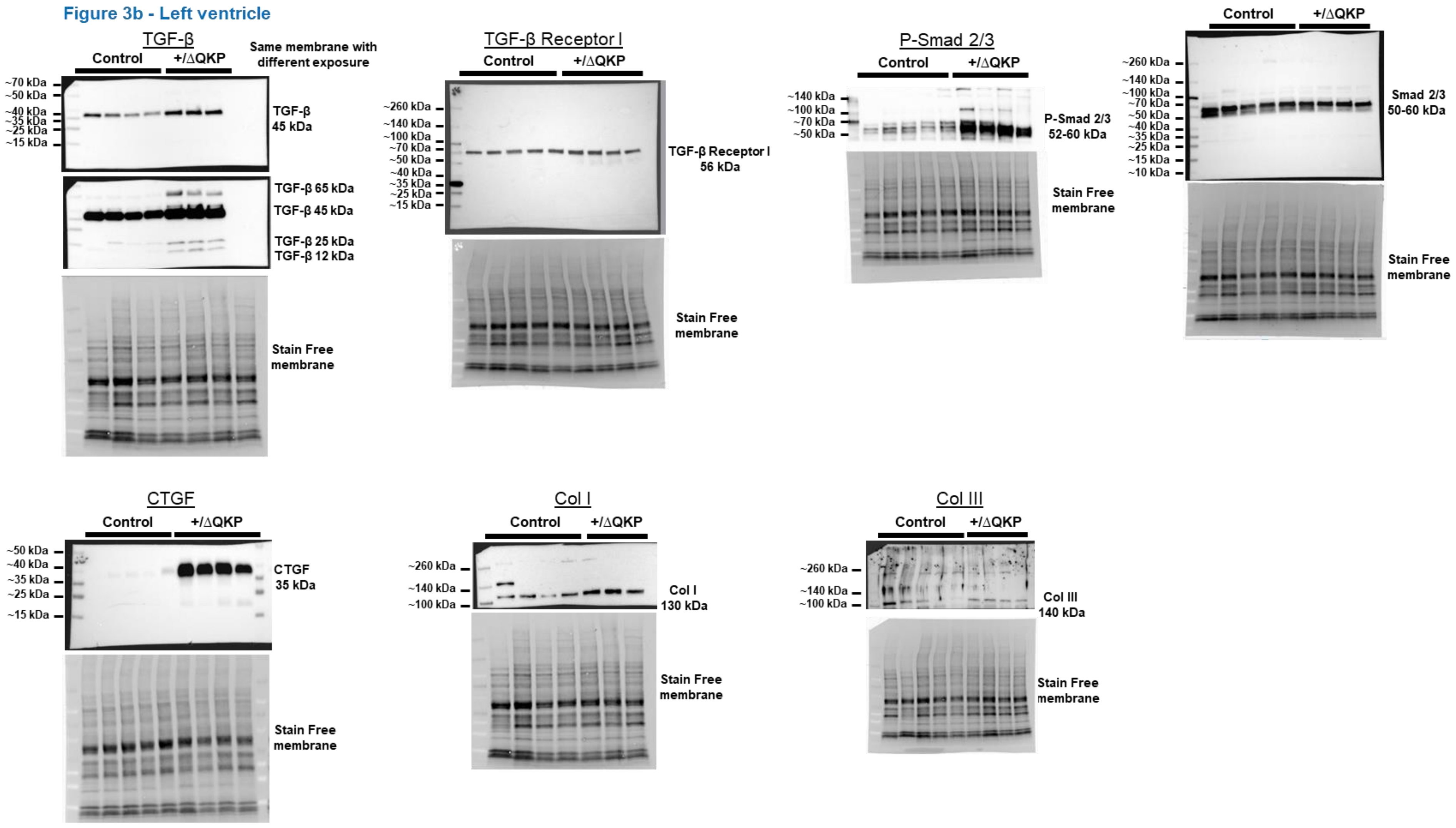

Supplementary Information 4. Uncropped and unmodified blot images from Figure 3b

Uncropped and unmodified blot images for the representative western blots of TGF-β, TGF- β receptor 1, Smad 2/3 and its phosphorylated form (P-Smad 2/3), connective tissue growth factor (CTGF), collagen I (Col I), and collagen III (Col III) in left ventricles from control and *Scn5a*$^{+/\Delta QKP}$ hearts.

Figure 4c

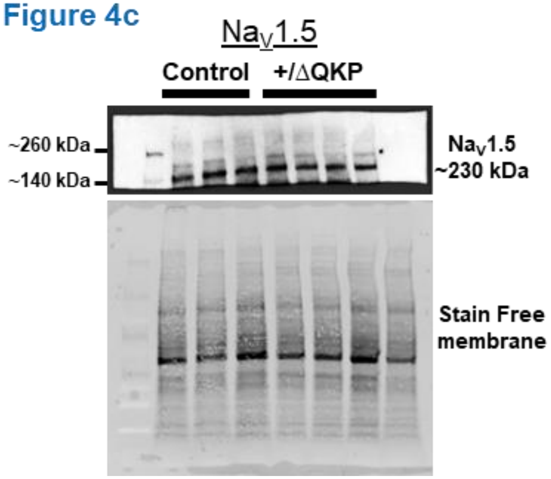

Supplementary Information 5. Uncropped and unmodified blot images from Figure 4c.

Uncropped and unmodified blot images for the representative western blots of Na$_v$1.in cardiac fibroblasts from control and *Scn5a*$^{+/\Delta QKP}$ mice.

Figure 5b

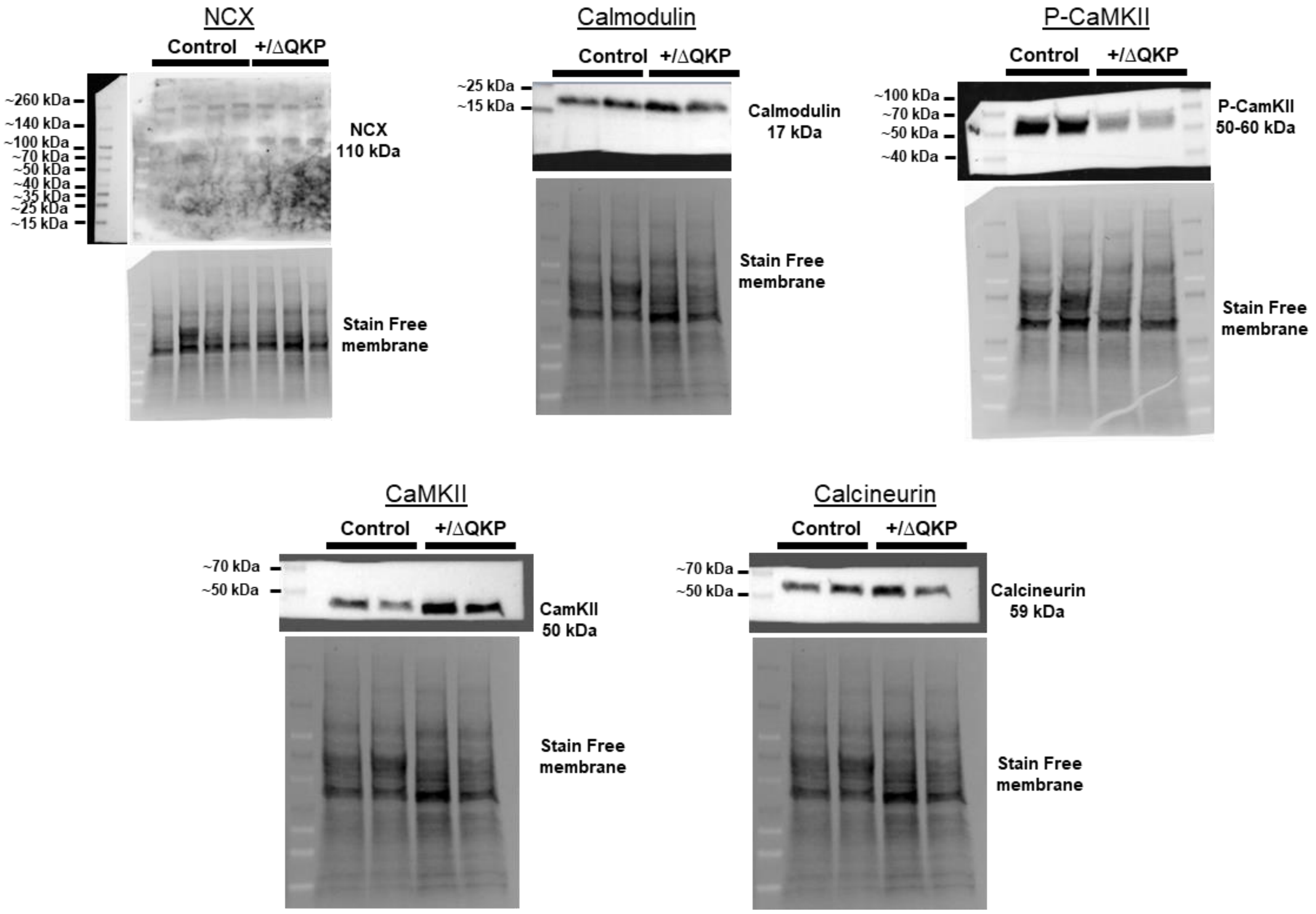

Supplementary Information 6. Uncropped and unmodified blot images from Figure 5b.

Uncropped and unmodified blot images for the representative western blots of NCX (sodium-calcium exchanger), calmodulin, phosphorylated-CamKII, CaMKII (Calcium/calmodulin-dependent protein kinase II), and calcineurin in fibroblasts from control and *Scn5a*$^{+/\Delta QKP}$ hearts.
