# Supplementary material for: Long QT syndrome type 3 gain-of-function of Nav1.5 increases ventricular fibroblasts proliferation and pro-fibrotic factors
Source: Commun Biol. 2025 Feb 11;8:216. doi: 10.1038/s42003-025-07636-5 (PMC11814334; doi:10.1038/s42003-025-07636-5)
Supplement: Supplementary file 2 — Description of Additional Supplementary Materials [file 42003_2025_7636_MOESM2_ESM.pdf]

Description of Additional Supplementary Files

File name: Supplementary Data 1

Description: Raw data for the graphs in the figures
