# Supplementary material for: Long QT syndrome type 3 gain-of-function of Nav1.5 increases ventricular fibroblasts proliferation and pro-fibrotic factors
Source: Commun Biol. 2025 Feb 11;8:216. doi: 10.1038/s42003-025-07636-5 (PMC11814334; doi:10.1038/s42003-025-07636-5)
Supplement: Supplementary file 4 — Reporting Summary [file 42003_2025_7636_MOESM4_ESM.pdf]

nature portfolio

Corresponding author(s): Derangeon

Last updated by author(s): 2024/12/17

Reporting Summary

Nature Portfolio wishes to improve the reproducibility of the work that we publish. This form provides structure for consistency and transparency in reporting. For further information on Nature Portfolio policies, see our Editorial Policies and the Editorial Policy Checklist.

Statistics

For all statistical analyses, confirm that the following items are present in the figure legend, table legend, main text, or Methods section.

n/a	Confirmed	
☐	☒	The exact sample size (*n*) for each experimental group/condition, given as a discrete number and unit of measurement
☐	☒	A statement on whether measurements were taken from distinct samples or whether the same sample was measured repeatedly
☐	☒	The statistical test(s) used AND whether they are one- or two-sided<br>*Only common tests should be described solely by name; describe more complex techniques in the Methods section.*
☐	☒	A description of all covariates tested
☐	☒	A description of any assumptions or corrections, such as tests of normality and adjustment for multiple comparisons
☐	☒	A full description of the statistical parameters including central tendency (e.g. means) or other basic estimates (e.g. regression coefficient) AND variation (e.g. standard deviation) or associated estimates of uncertainty (e.g. confidence intervals)
☐	☒	For null hypothesis testing, the test statistic (e.g. F, t, r) with confidence intervals, effect sizes, degrees of freedom and P value noted<br>*Give P values as exact values whenever suitable.*
☒	☐	For Bayesian analysis, information on the choice of priors and Markov chain Monte Carlo settings
☐	☒	For hierarchical and complex designs, identification of the appropriate level for tests and full reporting of outcomes
☐	☒	Estimates of effect sizes (e.g. Cohen's *d*, Pearson's *r*), indicating how they were calculated

Our web collection on statistics for biologists contains articles on many of the points above.

Software and code

Policy information about availability of computer code

Data collection	NA
Data analysis	NA

For manuscripts utilizing custom algorithms or software that are central to the research but not yet described in published literature, software must be made available to editors and reviewers. We strongly encourage code deposition in a community repository (e.g. GitHub). See the Nature Portfolio guidelines for submitting code & software for further information.

Data

Policy information about availability of data

All manuscripts must include a data availability statement. This statement should provide the following information, where applicable:

- Accession codes, unique identifiers, or web links for publicly available datasets
- A description of any restrictions on data availability
- For clinical datasets or third party data, please ensure that the statement adheres to our policy

The source data used to generate the graphs in the main figures are provided as Supplementary Information 2. Uncropped blot images of this study are provided in the Supplementary Information 2. The data from this study are available from the corresponding author upon reasonable request.

Research involving human participants, their data, or biological material

Policy information about studies with human participants or human data. See also policy information about sex, gender (identity/presentation), and sexual orientation and race, ethnicity and racism.

Reporting on sex and gender	NA
Reporting on race, ethnicity, or other socially relevant groupings	NA
Population characteristics	NA
Recruitment	NA
Ethics oversight	NA

Note that full information on the approval of the study protocol must also be provided in the manuscript.

Field-specific reporting

Please select the one below that is the best fit for your research. If you are not sure, read the appropriate sections before making your selection.

☒ Life sciences ☐ Behavioural & social sciences ☐ Ecological, evolutionary & environmental sciences

For a reference copy of the document with all sections, see nature.com/documents/nr-reporting-summary-flat.pdf

Life sciences study design

All studies must disclose on these points even when the disclosure is negative.

Sample size	No statistical methods were used to determine the sample size.
Data exclusions	No data were excluded.
Replication	At least 2-3 replicates were used per biological experiments.
Randomization	Randomization was done at the time of fibroblats isolation for culture.
Blinding	Blinding was not possible since knock-in mice exhibited hypertrophied hearts compared to wildtype mice. However, immunostainings have been performed by a blinded scientist.

Reporting for specific materials, systems and methods

We require information from authors about some types of materials, experimental systems and methods used in many studies. Here, indicate whether each material, system or method listed is relevant to your study. If you are not sure if a list item applies to your research, read the appropriate section before selecting a response.

Materials & experimental systems

n/a	Involved in the study	
☐	☒	Antibodies
☒	☐	Eukaryotic cell lines
☒	☐	Palaeontology and archaeology
☐	☒	Animals and other organisms
☒	☐	Clinical data
☒	☐	Dual use research of concern
☒	☐	Plants

Methods

n/a	Involved in the study	
☒	☐	ChIP-seq
☒	☐	Flow cytometry
☒	☐	MRI-based neuroimaging

Antibodies

Antibodies used	Vimentin (Cell Signaling Ref 5741; 1: 1000), Cleaved caspase 3 (Cell Signaling Ref 9664; 1: 250), Caspase 3 (Cell Signaling, Ref 9662; 1: 250), Mcl-1 (Cell Signaling Ref 5453BC; 1: 500), α-SMA (Sigma-Aldrich, Ref A5228; 1: 1000), Periostin (Abcam, Ref ab14041; 1: 1000), Connexin 43 (Sigma Aldrich Ref C6219; 1: 10000), TGF-β (Cell Signaling Ref 3711S; 1: 500), CTGF (Santa Cruz Ref 1439 ; 1: 500),

	Smad-2/3 (Millipore Ref 07-408; 1: 250), P-Smad-2/3 (Cell Signaling Ref 8828S; 1: 250), Collagen Type I (Cell Signaling Ref 84336; 1: 500), or Collagen Type III (Rockland Ref 600-401-105; 1: 500)
Validation	The antibodies have been validated and published in two studies we have already conducted : DOI: 10.1016/j.phrs.2020.104922 and DOI: 10.1093/cvr/cvx026

Animals and other research organisms

Policy information about studies involving animals; ARRIVE guidelines recommended for reporting animal research, and Sex and Gender in Research

Laboratory animals	Mic; Scn5a+/ΔQKP mouse model was generated in PolyGene AG , Strain : C57BL6 ; Age : 2 and 4-week-old mice
Wild animals	NA
Reporting on sex	For this study, we used identical numbers of males and females without distinction, as no difference between the two sexes was observed
Field-collected samples	NA
Ethics oversight	Animal experiments were performed in the animal facility of Nantes University Health Research Institute (UTE – IRS-UN) which has been accredited by the French Ministry of Agriculture. The experimental procedures were approved by the regional ethic committee (CEEA – Pays de la Loire, France) according to the Directive 2010/63/EU of the European Union.

Note that full information on the approval of the study protocol must also be provided in the manuscript.

Plants

Seed stocks	NA
Novel plant genotypes	NA
Authentication	NA
